# Risco Cardiometabólico em Crianças e Adolescentes: O Paradoxo entre Índice de Massa Corporal e Aptidão Cardiorrespiratória

**DOI:** 10.36660/abc.20210593

**Published:** 2022-04-25

**Authors:** Luciana Tornquist, Debora Tornquist, Letícia B. Schneiders, Silvia I. R. Franke, Jane D. P. Renner, Cézane P. Reuter

**Affiliations:** 1 Programa de Pós-graduação em Educação Física Universidade Federal de Pelotas Pelotas RS Brasil Programa de Pós-graduação em Educação Física, Universidade Federal de Pelotas (UFPel), Pelotas, RS – Brasil; 2 Programa de Pós-graduação em Ciências do Movimento Humano Universidade Federal do Rio Grande do Sul Porto Alegre RS Brasil Programa de Pós-graduação em Ciências do Movimento Humano, Universidade Federal do Rio Grande do Sul (UFRGS), Porto Alegre, RS – Brasil; 3 Departamento de Ciências da Saúde Programa de Pós-graduação em Promoção da Saúde Universidade de Santa Cruz do Sul Santa Cruz do Sul RS Brasil Departamento de Ciências da Saúde, Programa de Pós-graduação em Promoção da Saúde, Universidade de Santa Cruz do Sul (UNISC), Santa Cruz do Sul, RS – Brasil

**Keywords:** Estudantes, Criança, Adolescente, Obesidade, Aptidão Cardiorespiratória, Metabolismo, Fatores de Risco

## Abstract

**Fundamento:**

Foi demonstrado que o risco cardiometabólico está inversamente associado à aptidão cardiorrespiratória (APCR) e positivamente associado ao índice de massa corporal (IMC).

**Objetivo:**

Analisar a associação de fatores de risco cardiometabólicos com IMC e APCR combinados em escolares de um município do sul do Brasil.

**Métodos:**

Estudo transversal com uma amostra de 1252 escolares de sete a 17 anos. Foram avaliados colesterol total (CT), HDL-c, LDL-c, triglicerídeos (TG), pressão arterial sistólica (PAS) e diastólica (PAD). APCR e IMC foram agrupados em uma variável e os escolares classificados como eutróficos/aptos, eutróficos/inaptos, excesso de peso/aptos e excesso de peso/inaptos. Análises foram realizadas por meio de Regressão de Poisson e uma alfa de 0,05 foi adotado.

**Resultados:**

Escolares classificados com excesso de peso/aptos demonstraram uma razão de prevalência (RP) de 1,50 (1,04 – 2,16) para TG alterado, 3,05 (2,05 – 4,54) para PAS e 2,70 (1,87 – 3,88) para PAD elevada. Escolares com excesso de peso/ inaptos apresentaram RP para CT alto de 1,24 (1,11 – 1,39) e 1,51 (1,11 – 2,04) para baixos níveis de HDL. Além disso, apresentaram um risco de 2,07 (1,60 – 2,69) para TG alterado, 3,26 (2,31 – 4,60) para PAS e 2,42 (1,76 – 3,32) para PAD elevada.

**Conclusão:**

O IMC apresentou um papel central na associação com o risco e a APCR demonstrou atenuar a associação entre fatores de risco e excesso de peso. Escolares com excesso de peso apresentaram um risco cardiometabólico mais elevado, mas o tamanho do efeito foi maior entre os inaptos.

## Introdução

O índice de massa corporal (IMC) e a aptidão cardiorrespiratória (APCR) têm sido associados de forma independente e opostas à maior ocorrência de risco cardiometabólico em crianças e adolescentes.^1^ No entanto, a relação conjunta dessas variáveis com o risco ainda não está clara, mas as evidências indicam que a APCR poderia atenuar a associação entre o excesso de peso e os fatores de risco cardiometabólicos.^4^

Nesse sentido, as evidências sugerem que sujeitos com sobrepeso e obesidade, mas com bons níveis de aptidão cardiorrespiratória, apresentam perfil cardiometabólico mais favorável do que sujeitos com excesso de adiposidade, mas baixos níveis de APCR.^1 , 5^ Também há indícios de que níveis mais elevados de APCR estão relacionados a menor risco de mortalidade entre grupos com IMC semelhante^6^ e que níveis satisfatórios de APCR na infância podem mitigar riscos cardiometabólicos relacionados ao sobrepeso e obesidade na vida adulta.^7^

O paradoxo de indivíduos obesos, mas com bons níveis de APCR que não apresentam risco significativo para fatores cardiometabólicos, já foi evidenciado em adultos.^8 , 9^ Em crianças e adolescentes, esse paradoxo ainda é inconsistente.^9 , 10^ Diante dessas premissas, o objetivo do presente estudo é analisar a associação dos fatores de risco cardiometabólicos com o IMC e a APCR combinados em escolares de um município do sul do Brasil. Nossa hipótese é que escolares com sobrepeso e obesos, mas com boa aptidão cardiorrespiratória, apresentarão menor risco do que escolares com IMC semelhante, mas baixos níveis de aptidão.

## Métodos

Estudo transversal com base nos dados da pesquisa “Saúde do Escolar – Fase II”, aprovada pelo Comitê de Ética em Pesquisa com Seres Humanos local, protocolo 3044/11. Para participar da pesquisa, as crianças e adolescentes precisaram apresentar o Termo de Consentimento Livre e Esclarecido (TCLE) assinado pelos responsáveis.

Os critérios de inclusão estabelecidos para o estudo foram: pertencer à faixa etária de 7 a 17 anos; não ter contraindicação para coleta de amostra biológica (sangue), não ter qualquer limitação para a realização de testes de aptidão física. Foram excluídos do estudo os alunos que não preencheram corretamente os instrumentos de investigação, não realizaram coleta de sangue ou teste de aptidão física.

As coletas de dados foram realizadas em 2011 e 2012 no campus da universidade, em dia e horário previamente agendados pelos pesquisadores com a escola. O cálculo amostral foi realizado para regressão de Poisson, por meio do programa G * Power 3.1 (Heinrich-Heine-Universität – Düsseldorf, Alemanha), considerando um poder de teste (1 - β) = 0,95, nível de significância α = 0,05, e um tamanho do efeito de 0,30.

**Figura 1:**
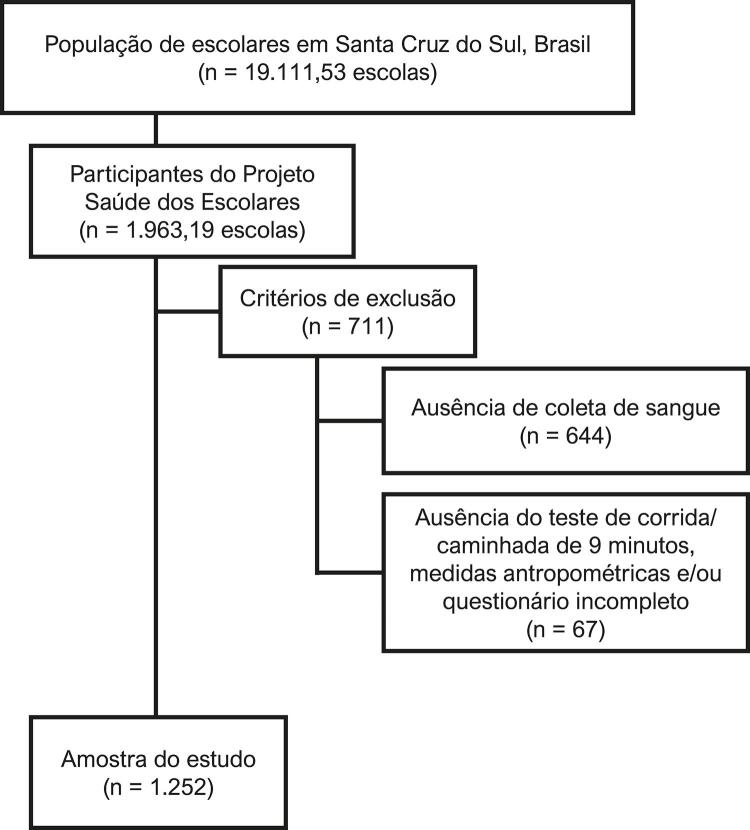
– Fluxograma do processo de selação da amostra.

A seleção dos sujeitos que compuseram a amostra ocorreu de forma aleatória, com as escolas selecionadas estratificadas por área urbana e rural. A área urbana foi estratificada por centro e periferia (sul, norte, leste e oeste) e a rural por regiões sul, norte, leste e oeste. Aplicados os critérios de exclusão, a amostra do presente estudo é composta por 1252 escolares pertencentes a 19 escolas do município de Santa Cruz do Sul (RS, Brasil). A figura 1 apresenta o fluxograma com o processo de seleção amostral.

Foram realizadas medidas de peso e altura no início da manhã, com o sujeito em jejum e vestindo roupas leves e descalço. A partir dessas medidas, o IMC foi calculado utilizando a fórmula IMC = peso / altura^2^ (kg/m^2^) e classificado de acordo com as curvas de percentil do CDC / NCHS,^11^ segundo sexo e idade, considerando baixo peso (<p5), eutrófico (≥p5 e <p85), sobrepeso (p≥85 e <p95) e obesidade (≥p95).

A aptidão cardiorrespiratória foi avaliada por meio do teste de corrida e caminhada de 9 minutos realizado em pista de atletismo, conforme protocolo e pontos de corte para sexo e idade do manual Projeto Esporte Brasil (PROESP-BR).^12^ O manual preconiza que para a realização do teste os alunos devem correr / caminhar a maior distância possível durante o tempo de nove minutos, sem pausas no período. Ao final, a distância percorrida pelos escolares (em metros) foi classificada considerando os valores críticos propostos pelo manual para idade e sexo.

Uma variável combinada foi gerada a partir das categorias de IMC e APCR, utilizada como exposição no presente estudo. Essa variável foi classificada em quatro categorias: (1) eutróficos/ aptos: escolares com baixo peso e eutróficos e classificados como aptos na avaliação da APCR; (2) Eutróficos/ inaptos: escolares com baixo peso e eutróficos e classificados como inaptos na avaliação da APCR; (3) excesso de peso/ aptos: escolares classificados com sobrepeso ou obesidade e como aptos na avaliação da APCR; (4) excesso de peso/ inaptos: escolares classificados com sobrepeso ou obesidade e inaptos.

Os desfechos avaliados foram os fatores de risco cardiometabólicos: colesterol total (CT), colesterol HDL (HDL-c), colesterol LDL (LDL-c), triglicerídeos (TG), pressão arterial sistólica (PAS) e diastólica (PAD). As variáveis bioquímicas foram avaliadas por meio da coleta de sangue realizada na veia braquial, após jejum de 12 horas. As análises de CT, TG e HDL-c foram realizadas em amostra de soro, em equipamento automatizado Miura One (I.S.E, Roma, Itália), utilizando kits comerciais DiaSys (Diagnostic Systems, Alemanha). Para a determinação do LDL-c, foi utilizado o cálculo LDL = CT - HDL-c - (Triglicerídeos/5) de acordo com a fórmula de Friedewald, Levy e Fredrickson.^13^ Os níveis de lipídios séricos dos alunos foram classificados de acordo com os pontos de corte do National Heart Lung and Blood Institute.^14^

Pressão arterial foi aferida com base no método auscultatório, utilizando-se esfigmomanômetro para perímetro braquial e estetoscópio colocado no braço esquerdo. O aluno ficava sentado, em repouso mínimo de 5 minutos. A classificação da PAS e PAD foi realizada de acordo com as VI Diretrizes Brasileiras de Hipertensão Arterial.^15^

As variáveis sexo, idade, local de moradia, tipo de escola, classe econômica e atividade física foram coletadas por meio de questionário e utilizadas como variáveis de controle no presente estudo. A partir das idades informadas, a amostra foi classificada em duas faixas etárias: (1) crianças: de 7 a 12 anos e (2) adolescentes: de 13 a 17 anos.

A classe econômica dos escolares foi classificada pelo critério da ABEP.^16^ A partir dessa classificação, as classes econômicas foram agrupadas em alta – classes A1, A2, B1 e B2; (2) intermediária – classes C1 e C2 e (3) inferior – classes D e E. A prática de atividade física (AF) foi investigada a partir da questão “Você pratica atualmente algum esporte/ atividade física?”. Os escolares foram instruídos a relatar apenas atividades físicas realizadas no lazer, não contabilizando atividades realizadas em aulas de educação física, deslocamentos, laborais ou domésticas. Os escolares foram classificados em (1) ativos: escolares que praticam algum esporte ou atividade física e (2) inativos: escolares que relataram não praticar nenhuma atividade.

### Análises estatísticas

As análises estatísticas foram realizadas no programa SPSS v.23.0 (IBM SPSS Statistics for Windows, IBM Corp., NY, USA). Primeiramente, foram realizadas análises descritivas de frequências simples e relativas da amostra quanto às características de sexo, faixa etária, classe econômica, tipo de escola, local de moradia, prática de atividade física e fatores de risco cardiometabólico (CT, HDL-c, LDL-c, TG, PAS e PAD), de acordo com as categorias da variável IMC/APCR. O teste do qui-quadrado de Pearson foi usado para essas comparações. A idade da amostra foi descrita através de média e desvio padrão.

Regressão de Poisson com estimativa robusta foi empregada para calcular as razões de prevalência (RP) brutas e ajustadas e seus respectivos intervalos de confiança (IC95%) dos fatores de risco cardiometabólicos de acordo com a variável independente IMC/ APCR. Para as análises ajustadas, as variáveis sexo, idade, classe econômica, tipo de escola, local de moradia e prática de AF foram testadas para cada desfecho, sendo adotado um p ≤ 0,20 para definir a entrada da variável no modelo. Para todos os modelos ajustados finais, o nível de significância obtido foi <0,001. Para todas as análises, o alfa adotado foi de 5%.

## Resultados

Um total de 1.252 alunos foram incluídos no estudo. A média de idade de foi 11,88 ± 3,02 anos, a maioria é do sexo masculino, adolescentes e residem na zona urbana do município ( Tabela 1 ). A taxa de inatividade física da amostra é de 36,5%. A taxa de sobrepeso e obesidade é de 29,0% e 50,8% tinham baixos níveis de aptidão cardiorrespiratória (dados não mostrados). As maiores prevalências de escolares com sobrepeso/ obesidade e baixa aptidão física foram encontradas entre os adolescentes, do sexo feminino e residentes na zona urbana.

**Tabela 1:** – Características da amostra de acordo com o IMC e a APCR dos escolares na faixa etária de 7 aos 17 anos do município de Santa Cruz do Sul (RS - Brasil), 2011-2012 (n= 1.252)

	Eutrófico/ apto n (%)	Eutrófico/ inapto n (%)	Excesso de peso/ apto n (%)	Excesso de peso/ inapto n (%)	Total n (%)	p*
**Sexo**						**< 0,001**
Masculino	229 (47,2)	77 (58,8)	150 (37,1)	111 (47,8)	567 (45,3)	
Feminino	256 (52,8)	54 (41,2)	254 (62,9)	121 (52,2)	685 (54,7)	
**Faixa etária**						**< 0,001**
Criança	140 (28,9)	54 (41,2)	63 (15,6)	85 (36,6)	342 (27,3)	
Adolescente	345 (71,1)	77 (58,8)	341 (84,4)	147 (63,4)	910 (72,7)	
**Zona de moradia**						**0,004**
Urbana	255 (52,6)	73 (55,7)	253 (62,6)	149 (64,2)	730 (58,3)	
Rural	230 (47,4)	58 (44,3)	151 (37,4)	83 (35,8)	522 (41,7)	
**Classe econômica**						**0,480**
Alta (A – B)	255 (52,6)	69 (52,7)	226 (55,9)	129 (55,6)	679 (54,2)	
Média (C)	217 (44,7)	55 (42,0)	170 (42,1)	95 (40,9)	537 (42,9)	
Baixa (D – E)	13 (2,7)	7 (5,3)	8 (2,0)	8 (3,4)	36 (2,9)	
**Tipo de escola**						**0,683**
Pública	453 (93,4)	121 (92,4)	380 (94,1)	221 (95,3)	1175 (93,8)	
Privada	32 (6,6)	10 (7,6)	24 (5,9)	11 (4,7)	77 (6,2)	
**Atividade Física**						**0,004**
Ativo	335 (69,1)	87 (66,4)	239 (59,2)	134 (57,8)	795 (63,5)	
Inativo	150 (30,9)	44 (33,6)	165 (40,8)	98 (42,2)	457 (36,5)	

*Teste do qui-quadrado.

Com relação aos fatores de risco avaliados, as maioras prevalências são observadas para os níveis elevados de CT e LDL-c. Para todos os fatores de risco, exceto LDL-c e PAD, as maiores prevalências foram observadas entre estudantes com sobrepeso ou obesidade e baixa aptidão ( Tabela 2 ).

**Tabela 2:** – Fatores de risco cardiometabólicos segundo o IMC e a APCR dos escolares na faixa etária de 7 a 17 anos do município de Santa Cruz do Sul (RS - Brasil), 2011-2012 (n = 1,252)

	Eutrófico/ apto n (%)	Eutrófico/ inapto n (%)	Excesso de peso/ apto n (%)	Excesso de peso/ inapto n (%)	Total n (%)	p*
**Colesterol total**						**< 0,001**
Normal	214 (44,1)	51 (38,9)	176 (43,6)	66 (28,4)	507 (40,5)	
Alterado	271 (55,9)	80 (61,1)	228 (56,4)	166 (71,6)	745 (59,5)	
**HDL-c**						**0,021**
Normal	404 (83,3)	107 (81,7)	347 (85,9)	177 (76,3)	1035 (82,7)	
Alterado	81 (16,7)	24 (18,3)	57 (14,1)	55 (23,7)	217 (17,3)	
**LDL-c**						**0,025**
Normal	272 (56,1)	68 (51,9)	256 (63,4)	124 (53,4)	720 (57,5)	
Alterado	213 (43,9)	63 (48,1)	148 (36,6)	108 (46,6)	532 (42,5)	
**Triglecerídeos**						**< 0,001**
Normal	403 (83,1)	99 (75,6)	335 (82,9)	149 (64,2)	986 (78,8)	
Alterado	82 (16,9)	32 (24,4)	69 (17,1)	83 (35,8)	266 (21,2)	
**PAS**						**< 0,001**
Normal	441 (90,9)	104 (79,4)	354 (87,6)	178 (76,7)	1077 (86,0)	
Alterada	44 (9,1)	27 (20,6)	50 (12,4)	54 (23,3)	175 (14,0)	
**PAD**						**< 0,001**
Normal	428 (88,2)	99 (75,6)	348 (86,1)	178 (76,7)	1053 (84,1)	
Alterada	57 (11,8)	32 (24,4)	56 (13,9)	54 (23,3)	199 (15,9)	

HDL-c:Lipoproteína de alta densidade; LDL-c: Lipoproteína de baixa densidade; PAS: Pressão arterial sistólica; PAD: Pressão arterial diastólica.*Teste qui-quadrado.

A Tabela 3 mostra as razões de prevalência brutas e ajustadas para os fatores de risco cardiometabólicos segundo o IMC e APCR, sendo utilizados como referência os escolares eutróficos/aptos. Escolares com sobrepeso e obesidade apresentaram maior prevalência de taxas de triglicerídeos aumentadas e níveis de pressão arterial sistólica elevada, sendo essa prevalência maior entre os não aptos. A prevalência de taxas alteradas de TG foi 50% maior entre escolares excesso de peso/ aptos e 107% entre escolares excesso de peso/ inaptos.

**Tabela 3:** – Razões de prevalência brutas e ajustadas dos fatores de risco cardiometabólicos de acordo com IMC e APCR dos escolares na faixa etária de 7 a 17 anos do município de Santa Cruz do Sul (RS - Brasil), 2011-2012 (n = 1.252)

	Eutrófico/ inapto RP (IC95%)	Excesso de peso/ apto RP (IC95%)	Excesso de peso/ inapto RP (IC95%)	p*
**Colesterol total**				
Normal	1	1	1	
Alterado bruto	1,01 (0,90 – 1,14)	1,09 (093 – 1,28)	1,28 (1,14 – 1,43)	< 0,001
Alterado ajustado†	0,99 (0,88 – 1,11)	1,09 (0,93 – 1,28)	1,24 (1,11 – 1,39)	< 0,001
**HDL-c**				
Normal	1	1	1	
Alterado bruto	0,85 (0,62 – 1,15)	1,10 (0,73 – 1,66)	1,42 (1,05 – 1,93)	0,019
Alterado ajustado‡	0,81 (0,60 – 1,11)	1,19 (0,78 – 1,80)	1,51 (1,11 – 2,04)	0,003
**LDL-c**				
Normal	1	1	1	
Altered crude	0,83 (0,71 – 0,98)	1,10 (0,89 – 1,34)	1,06 (0,89 – 1,26)	0,030
Alterado ajustado§	0,85 (0,73 – 1,00)	1,17 (0,96 – 1,43)	1,14 (0,97 – 1,33)	0,005
**Triglecerídeos**				
Normal	1	1	1	
Alterado bruto	1,01 (0,76 – 1,35)	1,45 (1,01 – 2,07)	2,12 (1,63 – 2,75)	< 0,001
Alterado ajustado//	0,98 (0,74 – 1,32)	1,50 (1,04 – 2,16)	2,07 (1,60 – 2,69)	< 0,001
**PAS**				
Normal	1	1	1	
Alterado bruto	1,36 (0,93 – 2,00)	2,27 (1,47 – 3,52)	2,57 (1,78 – 3,70)	< 0,001
Alterada ajustada¶	1,19 (0,82 – 1,72)	3,05 (2,05 – 4,54)	3,26 (2,31 – 4,60)	< 0,001
**PAD**				
Normal	1	1	1	
Alterada bruta	1,02 (0,98 – 1,06)	1,11 (1,04 – 1,19)	1,10 (1,05 – 1,16)	< 0,001
Alterada ajustada#	1,04 (0,75 – 1,46)	2,70 (1,87 – 3,88)	2,42 (1,76 – 3,32)	< 0,001

HDL-c: Lipoproteína de alta densidade; LDL-c: Lipoproteína de baixa densidade; PAS: Pressão arterial sistólica; PAD: Pressão arterial diastólica.*Regressão de Poisson. Ajustado: †Sexo, faixa etária e região de moradia; ‡Idade contínua, região de moradia e tipo de escola; §Sexo, faixa etária, classe econômica, região de moradia, atividade física e tipo de escola; //Sexo, faixa etária, atividade física e tipo de escola; ¶Sexo, idade contínua e atividade física; #Sexo, idade contínua, atividade física e tipo de escola.

Escolares classificados com excesso de peso/ aptos e excesso de peso/ inaptos apresentaram uma prevalência duas vezes maior para PAS elevada. Escolares com sobrepeso e obesidade também apresentaram maior risco para PAD elevada, tanto aptos quanto inaptos. Em adicional, apenas escolares com excesso de peso e baixa aptidão física apresentaram risco para CT e HDL-c alterados, com um risco de 24% para colesterol elevado e 51% para baixo HDL-c.

## Discussão

Nossos achados demonstram que escolares com sobrepeso e obesidade apresentaram maior risco cardiometabólico, quando comparados aos escolares eutróficos e com bons níveis de aptidão física. Escolares eutróficos e com baixa aptidão não apresentaram maior prevalência de risco. Porém, nos escolares com excesso de peso, embora o risco para níveis elevados de TG e pressão arterial tenha sido demonstrado em escolares aptos, o tamanho de efeito foi maior entre escolares inaptos. Além disso, apenas escolares excesso de peso/ inaptos apresentaram risco para CT elevado e baixos níveis de HDL-c.

Em nosso estudo, a APCR parece não estar associada de forma independente a ocorrência de fatores de risco entre os escolares avaliados. Embora alguns estudos tenham apontado associação entre menor APCR e maior risco cardiometabólico,^2 , 3^ os resultados mostram que entre escolares eutróficos e inaptos não há associação com fatores de risco. Por outro lado, em escolares com excesso de peso, aptos e inaptos, há um aumento na prevalência de risco, propondo um papel central do IMC nessas associações. Esses achados são confirmados em estudo semelhante que utilizou IMC e APCR combinados e demonstrou que o grupo eutrófico e boa aptidão física apresentou o menor escore para síndrome metabólica, enquanto o grupo com sobrepeso e inapto apresentaram o maior.^5^

Nossos achados demonstraram que escolares com baixa aptidão combinada com sobrepeso e obesidade apresentaram maior prevalência de risco para quase todas as variáveis, exceto para LDL-c e PAD. Outros estudos também mostraram um perfil lipídico mais favorável em crianças e adolescentes com menor IMC e boa aptidão física.^17^ Foi demonstrado que crianças e adolescentes eutróficos e baixa APCR não apresentavam níveis pressóricos e perfil lipídico mais favoráveis do que eutróficos com bons níveis de APCR^20^ e que crianças e adolescentes mais magros, mas menos aptos, têm um perfil cardiometabólico mais favorável do que seus pares mais pesados e com boa aptidão.^19^

Embora a relação entre baixa aptidão e risco não tenha sido demonstrada em indivíduos eutróficos, em indivíduos com sobrepeso/ obesidade os resultados indicam que há um aumento no risco, e indicando que APCR pode atenuar essa relação. Um estudo com adolescentes europeus constatou que a APCR pode mediar parcialmente cerca de 10% dessa relação, demonstrando que o risco relacionado ao excesso de peso pode ser parcialmente mitigado com a melhora dos níveis da APCR.^4^

Outros estudos também mostraram que bons níveis de APCR apresentaram um papel benéfico na compensação de risco em escolares com excesso de peso, sugerindo que níveis moderados a altos de APCR podem mitigar as consequências prejudiciais atribuídas ao excesso de adiposidade.^5 , 18^ Além disso, algumas evidências mostraram que, embora a APCR tenha uma associação inversa com os fatores de risco, após o ajuste para o IMC, as associações são atenuadas ou não são mais significativas, comprovando que o IMC tem uma influência importante na relação entre a APCR e os fatores de risco.^21^

Nosso trabalho tem alguns pontos fortes a considerar, como o tamanho da amostra, representativo e preciso da população de escolares do município de Santa Cruz do Sul, município de médio porte do sul do Brasil. Diferindo da maioria das investigações, realizadas em grandes centros urbanos. Destacamos a avaliação conjunta das variáveis IMC e APCR como variável de exposição, ainda pouco explorada, e os diversos fatores de risco utilizados como desfecho.

Como limitação é importante considerarmos a possível influência de fatores não mensurados, especialmente maturação sexual, fatores genéticos, dieta e outros fatores de estilo de vida, como o tempo sedentário, uma vez que o risco cardiometabólico é uma questão multifatorial. A utilização do IMC na avaliação da adiposidade e a avaliação dos níveis de APCR através de estimativas indiretas por teste de pista apresentam limitações, embora sejam amplamente utilizadas, principalmente em avaliações populacionais.

Destacamos as preocupantes prevalências encontradas em nosso estudo para inatividade física, sobrepeso e obesidade, inaptidão física e fatores de risco cardiometabólicos. Nossos resultados são importantes do ponto de vista clínico e de saúde pública porque demonstram que, embora o IMC desempenhe um papel central na relação com os fatores de risco, níveis adequados de aptidão cardiorrespiratória podem mitigar o risco em escolares com sobrepeso e obesidade e, portanto, melhorar os níveis da aptidão pode ser uma estratégia importante, independente da perda de peso.

Nesse contexto, são preocupantes os indícios de que, embora os níveis de APCR tenham se mantido estáveis na última década na população pediátrica, mais de 80% dessas crianças apresentavam baixos níveis de aptidão.^25^ Tornando-se imprescindível incentivar essa população a cumprir as recomendações para a prática de atividades físicas, haja vista a importante relação que níveis recomendados de AF têm com os melhores índices de APCR.^2^

A relevância de investir em estratégias que promovam melhorias na aptidão da população jovem é reforçada por evidências que indicam que bons níveis de APCR durante a infância resultam em um perfil cardiometabólico mais saudável na idade adulta^7^ e que indivíduos inaptos têm o dobro de risco de mortalidade, independentemente do IMC, quando comparados a indivíduos aptos e eutróficos.^26^

## Conclusão

O risco cardiometabólico em escolares com sobrepeso e obesidade pode ser mitigado parcialmente, embora não seja eliminado, por níveis satisfatórios de aptidão cardiorrespiratória. Os baixos níveis de APCR em escolares eutróficos não parecem estar diretamente relacionado ao risco. Nossos resultados contribuem com as evidências existentes, que sugerem um papel protetor do APCR, atenuando os efeitos deletérios da obesidade na saúde cardiometabólica.
