# Supplementary material for: Risco Cardiometabólico em Crianças e Adolescentes: O Paradoxo entre Índice de Massa Corporal e Aptidão Cardiorrespiratória
Source: Arq Bras Cardiol. 2022 Apr 25;119(2):236–43. [Article in Portuguese] doi: 10.36660/abc.20210593 (PMC9363058; doi:10.36660/abc.20210593)
Supplement: Translation

# Translation: Cardiometabolic Risk in Children and Adolescents: The Paradox between Body Mass Index and Cardiorespiratory Fitness

**Authors:** Luciana Tornquist, Debora Tornquist, Letícia B. Schneiders, Silvia I. R. Franke, Jane D. P. Renner, Cézane P. Reuter

**Affiliations:** 1 Brasil Programa de Pós-graduação em Educação Física, Universidade Federal de Pelotas (UFPel), Pelotas, RS – Brasil; 2 Brasil Programa de Pós-graduação em Ciências do Movimento Humano, Universidade Federal do Rio Grande do Sul (UFRGS), Porto Alegre, RS – Brasil; 3 Brasil Departamento de Ciências da Saúde, Programa de Pós-graduação em Promoção da Saúde, Universidade de Santa Cruz do Sul (UNISC), Santa Cruz do Sul, RS – Brasil

**Keywords:** Students, Child, Adolescent, Obesity, Cardiorespiratory Fitness, Risk Factors

## Abstract

**Background:**

Cardiometabolic risk has been shown to be inversely associated with cardiorespiratory fitness (CRF) and positively associated with body mass index (BMI).

**Objective:**

Our objective was to analyze the association of cardiometabolic risk factors with combined BMI and CRF in schoolchildren from a city in southern Brazil.

**Methods:**

Cross-sectional study with a sample of 1252 schoolchildren aged seven to 17 years. Total cholesterol (TC), HDL-c, LDL-c, triglycerides (TG), systolic (SBP) and diastolic blood pressure (DBP) were evaluated. CRF and BMI were grouped into one variable and the schoolchildren were classified as eutrophic/fit, eutrophic/unfit, overweight-obese/fit, and overweight-obese/unfit. Crude and adjusted analyzes were performed using Poisson Regression and an alpha of 0.05 was adopted.

**Results:**

Overweight-obese and fit schoolchildren showed a prevalence ratio (PR) of 1.50 (1.04 – 2.16) for altered TG, 3.05 (2.05 – 4.54) for elevated SBP, and 2.70 (1.87 – 3.88) for elevated DBP. Overweight-obese and unfit schoolchildren showed a PR for high TC of 1.24 (1.11 – 1.39) and 1.51(1.11 – 2.04) for low HDL levels. In addition, they had a risk of 2.07 (1.60 – 2.69) for altered TG, 3.36 (2.31 – 4.60) for elevated SBP and 2.42 (1.76 – 3.32) for altered DBP.

**Conclusion:**

BMI played a central role in the association with risk and CRF was shown to attenuate the association between risk factors and obesity. Overweight-obese children and adolescents had a higher cardiometabolic risk, but the effect size was larger among the unfit.

## Introduction

Body mass index (BMI) and cardiorespiratory fitness (CRF) have been independently and oppositely associated with a higher occurrence of cardiometabolic risk in children and adolescents.^1^ However, the joint relationship of these variables with risk is still unclear, but evidence indicates that CRF could attenuate the association between overweight and cardiometabolic risk factors.^4^

In this regard, evidence suggests that subjects with overweight and obesity but good levels of cardiorespiratory fitness have a more favorable cardiometabolic profile than subjects with excess adiposity but low levels of CRF.^1 , 5^ There is also evidence that higher levels of CRF are related to lower mortality risk among groups with similar BMI^6^ and that satisfactory levels of CRF in childhood may mitigate cardiometabolic risks related to overweight and obesity in adulthood.^7^

The paradox of obese individuals but with good levels of CRF who do not have significant risk for cardiometabolic factors has already been evidenced in adults.^8 , 9^ In children and adolescents, this paradox is still inconsistent.^9 , 10^ Given these premises, the objective of the present study is to analyze the association of cardiometabolic risk factors with BMI and CRF combined in schoolchildren from a city in southern Brazil. Our hypothesis is that overweight and obese schoolchildren with good cardiorespiratory fitness will present a lower risk than schoolchildren with similar BMI but low levels of fitness.

## Method

Cross-sectional study based on data from the research “School Health – Phase II”, approved by the local Ethics Committee for Research with Human Beings, protocol 3044/11. To participate in the research, children and adolescents needed to present the Informed Consent Form (ICF) signed by their guardians.

The inclusion criteria established for the study were: belonging to the age group of 7 to 17 years old; not having any contraindication for biological sample collection (blood), not having any limitation for physical fitness tests. The schoolchildren who did not fill out the research instruments correctly did not collect blood or did not perform physical fitness tests were excluded from the study.

Data collection was carried out in 2011 and 2012 at the university campus, on a day and time previously scheduled by the researchers with the school. Sample calculation was performed for Poisson regression, using the G * Power 3.1 program (Heinrich-Heine-Universität - Düsseldorf, Germany), considering a test power (1 - β) = 0.95, significance level α = 0.05, and an effect size of 0.30.

The selection of the subjects that made up the sample occurred randomly, with the selected schools stratified by urban and rural areas. The urban area was stratified by center and periphery (south, north, east, and west) and the rural area by south, north, east, and west regions. After applying the exclusion criteria, the sample for the present study consists of 1252 schoolchildren belonging to 19 schools in the city of Santa Cruz do Sul (RS, Brazil). Figure 1 presents the flowchart with the sample selection process.

**Figure 1:** – Sample selection flowchart.

Weight and height measurements were taken in the early morning, with the subject fasting and wearing light clothes and barefoot. From these measurements, BMI was calculated using the formula BMI = weight / height^2^(kg/m^2^) and classified according to the CDC/ NCHS percentile curves,^11^ according to sex and age, considering underweight (<p5), eutrophic (≥p5 and <p85), overweight (p≥85 and <p95), and obesity (≥p95).

Cardiorespiratory fitness was assessed using the 9-minute running and walking test performed on an athletics track, according to the protocol and cutoff points for sex and age of the Brazil Sport Project (PROESP-BR) manual.^12^ The manual recommends that the students run/walk the longest distance possible for nine minutes, no breaks over the period. In the end, the distance covered by the students (in meters) was classified considering the critical values proposed by the manual for age and sex.

A combined variable was generated from the BMI and CRF categories, used as exposure in the present study. This variable was classified into four categories: (1) eutrophic/fit: schoolchildren with low weight and eutrophic and classified as fit in the CRF evaluation; (2) Eutrophic/unfit: schoolchildren with low weight and eutrophic and classified as unfit in the CRF evaluation; (3) overweight - obese/fit: schoolchildren classified with overweight or obesity and as fit; (4) overweight – obese/unfit: schoolchildren classified with overweight or obesity and unfit.

The outcomes evaluated were cardiometabolic risk factors: total cholesterol (TC), HDL-cholesterol (HDL-c), LDL-cholesterol (LDL-c), triglycerides (TG), systolic (SBP) and diastolic blood pressure (DBP). Biochemical variables were assessed by blood sampling from the brachial vein after a 12-hour fast. The analyses of TC, TG, and HDL-c were performed in serum sample, in automated equipment Miura One (I.S.E, Rome, Italy), using commercial kits DiaSys (Diagnostic Systems, Germany). For LDL-c determination, the calculation LDL = TC - HDL-c - (Triglycerides/5) according to the Friedewald, Levy and Fredrickson formula was used.^13^ The serum lipid levels of the students were classified according to the cut-off points of the National Heart Lung and Blood Institute.^14^

Blood pressure was measured based on the auscultatory method, using a sphygmomanometer for brachial perimeter and a stethoscope placed on the left arm. The student was seated, resting for at least 5 minutes. The classification of SBP and DBP was performed according to the VI Brazilian Guidelines on Hypertension.^15^

The variables sex, age, housing area, type of school, economic class, and physical activity were collected through a questionnaire and used as control variables in this study. Based on the ages reported, the sample was classified into two age groups: (1) children: from 7 to 12 years and (2) adolescents: from 13 to 17 years

The schoolchildren’s economic class was classified based on the ABEP criterion.^16^ From this classification, the economic classes were grouped into upper – classes A1, A2, B1 and B2; (2) middle – classes C1 and C2, and (3) lower – classes D and E. The practice of physical activity (PA) was investigated based on the question “Do you currently practice any sport/physical activity? The students were instructed to report only physical activities performed in leisure, not counting activities performed in physical education classes, commuting, work or domestic. The students were classified as (1) active: students who practice some sport or physical activity and (2) inactive: students who reported not practicing any activity.

### Statistical analysis

Statistical analyses were performed using SPSS v.23.0 software (IBM SPSS Statistics for Windows, IBM Corp., NY, USA). First, descriptive analyses of simple and relative frequencies of the sample were performed regarding the characteristics of sex, age group, economic class, type of school, housing area, practice of PA, and cardiometabolic risk factors (TC, HDL-c, LDL-c, TG, SBP, and DBP), according to the categories of the BMI/CRF variable. Pearson’s chi-square test was used for these comparisons. The age of the sample was described using mean and standard deviation.

Poisson regression with robust estimation was used to calculate the crude and adjusted prevalence ratios (PR) and their respective confidence intervals (95%CI) of cardiometabolic risk factors according to the independent variable BMI/CRF. For the adjusted analyses, the variables sex, age, economic class, type of school, housing area, and PA practice were tested for each outcome, and a p ≤ 0.20 was adopted to define the variable entry in the model. For all final fitted models, the significance level obtained was <0.001. For all analyses, the alpha adopted was 5%.

## Results

A total of 1,252 students were included in the study. The mean age was 11.88 ± 3.02 years, most of them are male, teenagers and live in the urban area of the city ( Table 1 ).The physical inactivity rate of the sample is 36.5%. The rate of overweight and obesity is 29.0% and 50.8% had low levels of cardiorespiratory fitness (data not shown). The highest prevalences of schoolchildren with overweight/ obesity and low CRF were found among adolescents, girls, and urban area residents.

**Table 1:** – Characteristics of the sample according to BMI and CRF of schoolchildren aged 7 to 17 years in the municipality of Santa Cruz do Sul (RS - Brazil), 2011-2012 (n = 1,252)

	Eutrophic/ fit n (%)	Eutrophic/ unfit n (%)	Overweight- obese/ fit n (%)	Overweight-obese/ unfit n (%)	Total n (%)	p*
**Sex**						**< 0.001**
Male	229 (47.2)	77 (58.8)	150 (37.1)	111 (47.8)	567 (45.3)	
Female	256 (52.8)	54 (41.2)	254 (62.9)	121 (52.2)	685 (54.7)	
**Age range**						**< 0.001**
Child	140 (28.9)	54 (41.2)	63 (15.6)	85 (36.6)	342 (27.3)	
Adolescent	345 (71.1)	77 (58.8)	341 (84.4)	147 (63.4)	910 (72.7)	
**Housing area**						**0.004**
Urban	255 (52.6)	73 (55.7)	253 (62.6)	149 (64.2)	730 (58.3)	
Rural	230 (47.4)	58 (44.3)	151 (37.4)	83 (35.8)	522 (41.7)	
**Economic Class**						**0.480**
Upper (A – B)	255 (52.6)	69 (52.7)	226 (55.9)	129 (55.6)	679 (54.2)	
Middle (C)	217 (44.7)	55 (42.0)	170 (42.1)	95 (40.9)	537 (42.9)	
Lower (D – E)	13 (2.7)	7 (5.3)	8 (2.0)	8 (3.4)	36 (2.9)	
**School type**						**0.683**
Public	453 (93.4)	121 (92.4)	380 (94.1)	221 (95.3)	1175 (93.8)	
Private	32 (6.6)	10 (7.6)	24 (5.9)	11 (4.7)	77 (6.2)	
**Physical activity**						**0.004**
Active	335 (69.1)	87 (66.4)	239 (59.2)	134 (57.8)	795 (63.5)	
Inactive	150 (30.9)	44 (33.6)	165 (40.8)	98 (42.2)	457 (36.5)	

*Chi squared test

Regarding the assessed risk factors, the highest prevalence are observed for high levels of TC and LDL-c. For all risk factors except LDL-c and DBP, the highest prevalence’s were observed among students with overweight or obesity and low cardiorespiratory fitness ( Table 2 ).

**Table 2:** – Cardiometabolic risk factors according to BMI and CRF of schoolchildren aged 7 to 17 years in the municipality of Santa Cruz do Sul (RS - Brazil), 2011-2012 (n= 1,252)

	Eutrophic/ fit n (%)	Eutrophic/ unfit n (%)	Overweigh-obese/ fit n (%)	Overweight-obese/ unfit n (%)	Total n (%)	p*
**Cholesterol**						**< 0.001**
Normal	214 (44.1)	51 (38.9)	176 (43.6)	66 (28.4)	507 (40.5)	
Altered	271 (55.9)	80 (61.1)	228 (56.4)	166 (71.6)	745 (59.5)	
**HDL-c**						**0.021**
Normal	404 (83.3)	107 (81.7)	347 (85.9)	177 (76.3)	1035 (82.7)	
Altered	81 (16.7)	24 (18.3)	57 (14.1)	55 (23.7)	217 (17.3)	
**LDL-c**						**0.025**
Normal	272 (56.1)	68 (51.9)	256 (63.4)	124 (53.4)	720 (57.5)	
Altered	213 (43.9)	63 (48.1)	148 (36.6)	108 (46.6)	532 (42.5)	
**Triglycer-ides**						**< 0.001**
Normal	403 (83.1)	99 (75.6)	335 (82.9)	149 (64.2)	986 (78.8)	
Altered	82 (16.9)	32 (24.4)	69 (17.1)	83 (35.8)	266 (21.2)	
**SBP**						**< 0.001**
Normal	441 (90.9)	104 (79.4)	354 (87.6)	178 (76.7)	1077 (86.0)	
Altered	44 (9.1)	27 (20.6)	50 (12.4)	54 (23.3)	175 (14.0)	
**DBP**						**< 0.001**
Normal	428 (88.2)	99 (75.6)	348 (86.1)	178 (76.7)	1053 (84.1)	
Altered	57 (11.8)	32 (24.4)	56 (13.9)	54 (23.3)	199 (15.9)	

HDL-c: High-density lipoprotein cholesterol; LDL-c: Low-density lipoprotein cholesterol; SBP: Systolic blood pressure; DBP: Diastolic blood pressure. *Chi-squared test.

Table 3 shows the crude and adjusted prevalence ratios for cardiometabolic risk factors according to BMI and CRF, using eutrophic/ fit students as reference. Overweight and obese schoolchildren had a higher prevalence of increased triglyceride rates and elevated systolic blood pressure levels, and this prevalence was higher among the unfit. The prevalence of altered TG rates was 50% higher among overweight-obese/ fit schoolchildren and 107% among overweight-obese/unfit students. Schoolchildren classified with overweight/fit and overweight/unfit had a two-fold higher a prevalence of high SBP. Overweight and obese students were also at higher risk for high DBP, both fit and unfit. In addition, only students with overweight and low physical fitness were at risk for altered TC and HDL-c, with a risk of 24% for high cholesterol and 51% for low HDL-c.

**Table 3:** – Crude and adjusted prevalence ratios of cardiometabolic risk factors according to BMI and CRF of schoolchildren aged 7 to 17 years in the municipality of Santa Cruz do Sul (RS - Brazil), 2011-2012 (n = 1,252)

	Eutrophic// unfit PR (IC95%)	Overweight-obese/ fit PR (IC95%)	Overweight-obese/ unfit PR (IC95%)	p*
**Cholesterol**				
Normal	1	1	1	
Altered crude	1.01 (0.90 – 1.14)	1.09 (093 – 1.28)	1.28 (1.14 – 1.43)	< 0.001
Altered adjusted†	0.99 (0.88 – 1.11)	1.09 (0.93 – 1.28)	1.24 (1.11 – 1.39)	< 0.001
**HDL-c**				
Normal	1	1	1	
Altered crude	0.85 (0.62 – 1.15)	1.10 (0.73 – 1.66)	1.42 (1.05 – 1.93)	0.019
Altered adjusted‡	0.81 (0.60 – 1.11)	1.19 (0.78 – 1.80)	1.51 (1.11 – 2.04)	0.003
**LDL-c**				
Normal	1	1	1	
Altered crude	0.83 (0.71 – 0.98)	1.10 (0.89 – 1.34)	1.06 (0.89 – 1.26)	0.030
Altered adjusted§	0.85 (0.73 – 1.00)	1.17 (0.96 – 1.43)	1.14 (0.97 – 1.33)	0.005
**Triglycerides**				
Normal	1	1	1	
Altered crude	1.01 (0.76 – 1.35)	1.45 (1.01 – 2.07)	2.12 (1.63 – 2.75)	< 0.001
Altered adjusted//	0.98 (0.74 – 1.32)	1.50 (1.04 – 2.16)	2.07 (1.60 – 2.69)	< 0.001
**SBP**				
Normal	1	1	1	
Altered crude	1.36 (0.93 – 2.00)	2.27 (1.47 – 3.52)	2.57 (1.78 – 3.70)	< 0.001
Altered adjusted¶	1.19 (0.82 – 1.72)	3.05 (2.05 – 4.54)	3.26 (2.31 – 4.60)	< 0.001
**DBP**				
Normal	1	1	1	
Altered crude	1.02 (0.98 – 1.06)	1.11 (1.04 – 1.19)	1.10 (1.05 – 1.16)	< 0.001
Altered adjusted#	1.04 (0.75 – 1.46)	2.70 (1.87 – 3.88)	2.42 (1.76 – 3.32)	< 0.001

HDL-c: High-density lipoprotein cholesterol; LDL-c: Low-density lipoprotein cholesterol; SBP: Systolic blood pressure; DBP: Diastolic blood pressure. *Poisson regression. Adjusted: †Sex, age range and housing area. ‡Continuous age, housing area and school type. §Sex, age range, economic class, housing area, physical activity and school type. //Sex, age range, physical activity and school type. ¶Sex, continuous age and physical activity. #Sex, continuous age, physical activity and school type.

## Discussion

Our findings show that overweight and obese schoolchildren had a higher cardiometabolic risk when compared to eutrophic schoolchildren with good levels of physical fitness. Eutrophic students and those with low physical fitness did not present higher risk prevalence. However, in overweight schoolchildren, although the risk for elevated TG and blood pressure levels was demonstrated in fit schoolchildren, the effect size was greater among unfit schoolchildren. In addition, only overweight/unfit schoolchildren were at risk for elevated TC and low HDL-c levels.

In our study, CRF does not seem to be independently associated with the the occurrence of risk factors among the evaluated schoolchildren. Although some studies have pointed out an association between lower CRF and higher cardiometabolic risk,^2 , 3^ the results show that among eutrophic and unfit students there is no association with risk factors. On the other hand, in overweight, fit and unfit schoolchildren, there is an increase in risk prevalence, proposing a central role of BMI in these associations. These findings are confirmed in a similar study that used combined BMI and CRF and showed that the eutrophic and fit group had the lowest score for metabolic syndrome, while the overweight and unfit group had the highest.^5^

Our findings showed that schoolchildren with low fitness combined with overweight and obesity had higher prevalence of risk for almost all variables, except for LDL-c and DBP. Other studies also showed a more favorable lipid profile in children and adolescents with lower BMI and good fitness.^17^ It has been shown that eutrophic children and adolescents with low CRF did not have more favorable blood pressure levels and lipid profile than eutrophic children and adolescents with good CRF^20^ and that thinner but less fit children and adolescents have a more favorable cardiometabolic profile than their heavier peers with good cardiorespiratory fitness.^19^

Although the relationship between low fitness and risk has not been demonstrated in eutrophic individuals, in overweight/obese individuals the results indicate that there is an increase in risk, and indicating that CRF may mitigate this relationship. A study of European adolescents found that CRF can partially mediate about 10% of this relationship, demonstrating that overweight-related risk can be partially mitigated by improving CRF levels.^4^

Other studies have also shown that good levels of CRF showed a beneficial role in risk compensation in overweight schoolchildren, suggesting that moderate to high levels of CRF may mitigate the detrimental consequences attributed to excess adiposity.^5 , 18^ Furthermore, some evidence has shown that although CRF has an inverse association with risk factors, after adjustment for BMI, the associations are attenuated or are no longer significant, proving that BMI has an important influence on the relationship between CRF and risk factors.^21^

Our work has some strong points to consider, such as the sample size, representative of the schoolchildren population in Santa Cruz do Sul, a medium-sized municipality in the south of Brazil. Different from most investigations, carried out in large urban centers. We highlight the joint evaluation of BMI and CRF as an exposure variable, still little explored, and the several risk factors evaluated as outcome.

As a limitation, it is important to consider the possible influence of unmeasured factors, especially sexual maturation, genetic factors, diet, and other lifestyle factors such as sedentary time, since cardiometabolic risk is a multifactorial issue. The use of BMI in adiposity assessment and the assessment of CRF levels through indirect estimates by runway test have limitations, although they are widely used, especially in population assessments.

We highlight the worrying prevalence’s found in our study for physical inactivity, overweight and obesity, physical unfitness, and cardiometabolic risk factors. Our results are important from a clinical and public health perspective because they demonstrate that although BMI plays a central role in the relationship with risk factors, adequate levels of cardiorespiratory fitness can mitigate risk in overweight and obese schoolchildren, and therefore improving fitness levels may be an important strategy independent of weight loss.

In this sense, is worrying about the indications that, although the levels of CRF have remained stable in the last decade in the pediatric population, more than 80% of these children had low levels of fitness.^25^ It is essential to encourage this population to comply with the recommendations for physical activity, given the important relationship that recommended levels of PA have with better rates of CRF.^2^

The relevance of investing in strategies that promote improvements in fitness in the young population is reinforced by evidence indicating that good levels of CRF during childhood result in a healthier cardiometabolic profile in adulthood^7^ and that unfit individuals have twice the risk of mortality, regardless of BMI, when compared to fit and eutrophic individuals.^26^

## Conclusion

Cardiometabolic risk in overweight and obese schoolchildren can be partially mitigated, although not eliminated, by satisfactory levels of cardiorespiratory fitness. Low levels of CRF in eutrophic schoolchildren do not appear to be directly related to risk. Our results contribute to existing evidence suggesting a protective role of CRF, mitigating the deleterious effects of obesity on cardiometabolic health.
